# Cleaning and sampling protocol for analysis of mercury and dissolved organic matter in freshwater systems

**DOI:** 10.1016/j.mex.2018.08.002

**Published:** 2018-08-23

**Authors:** Andrea G. Bravo, Dolly N. Kothawala, Katrin Attermeyer, Emmanuel Tessier, Pascal Bodmer, David Amouroux

**Affiliations:** a Department of Environmental Chemistry, Institute of Environmental Assessment and Water Research (IDAEA), Spanish National Research Council (CSIC), Barcelona, Spain; b Limnology/Department of Ecology and Genetics, Uppsala University, Uppsala, Sweden; c CNRS/ UNIV PAU & PAYS ADOUR, Institut des Sciences Analytiques et de Physico-Chimie pour l’Environnement et les Materiaux, UMR5254, MIRA, Pau, France; d Institute for Environmental Sciences, University of Koblenz-Landau, Landau, Germany; e Chemical Analytics and Biogeochemistry, Leibniz-Institute of Freshwater Ecology and Inland Fisheries, Berlin, Germany

**Keywords:** Sampling protocol for total and methylmercury as well as dissolved organic matter analyses in freshwater ecosystems, Sampling, Preservation, Mercury, Methylmercury, Water, DOC, Fluorescence

## Abstract

Mercury (Hg), and in particular its methylated form (methylmercury, MeHg), is a hazardous substance with the potential to produce significant adverse neurological and other health effects. Enhanced anthropogenic emissions and long-range transport of atmospheric Hg have increased Hg concentrations above background levels in aquatic systems. In this context, the Minamata Convention, a global legally binding agreement that seeks to prevent human exposure to Hg, was signed and enforced by 128 countries, and today more than 90 Parties have ratified it. All these Parties have committed to develop Hg monitoring programs to report the effectiveness of the convention. For this purpose, we provide a standardized cleaning and water sampling protocol for the determination of total-Hg and MeHg in freshwaters at ambient levels. As Hg and organic matter are tightly bound, the protocol also describes sample collection for dissolved organic carbon (DOC) concentration and characterization of dissolved organic matter (DOM) composition by fluorescence spectroscopy. This protocol is highly useful to non-experts without a prior background in Hg sampling and analysis, and can serve as a useful basis for national monitoring programs. Furthermore, this protocol should help increase quantitative inventories of DOC, inorganic-Hg (IHg) and MeHg concentrations and DOM composition in freshwater, which are severely lacking at a global scale.

• *Provides a standardized method to collect water samples for IHg, MeHg, DOC and DOM composition from freshwater ecosystems.*

## Graphical abstract

**Figure:**
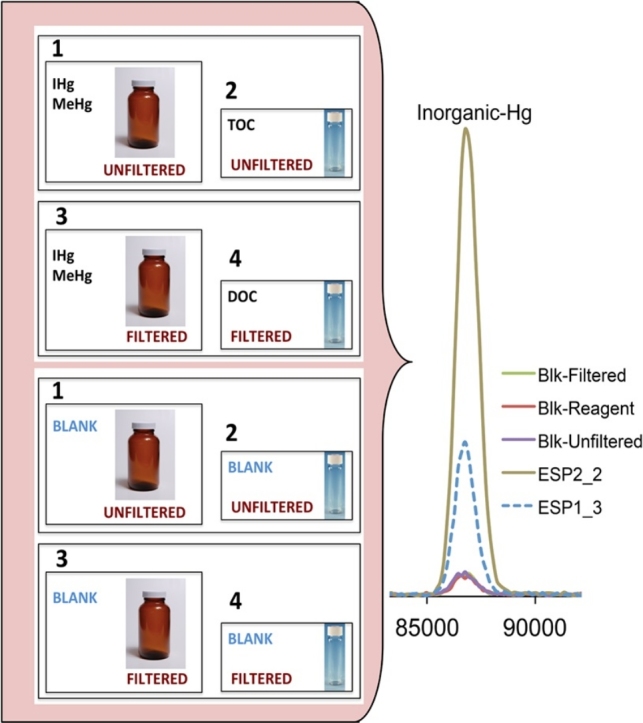

**Specifications Table**

**Table:** 

Subject area	• *Chemistry* • *Environmental Science*
More specific subject area	*Mercury Biogeochemistry in Aquatic Ecosystems*
Method name	*Sampling protocol for total and methylmercury as well as dissolved organic matter analyses in freshwater ecosystems*

## Methods details

Global standardized quantitative inventories of inorganic-Hg (IHg) and methylmercury (MeHg) concentrations in the aquatic network are currently lacking but are undoubtedly needed to report the effectiveness of the Minamata Convention. However, determining IHg and MeHg concentrations at low, but environmentally relevant levels remain challenging. Ambient low-level Hg samples are susceptible to contamination from many sources, including improperly cleaned equipment, improper sample-collection techniques, contaminated reagents, and atmospheric inputs from dust, dirt, and rain. Clean procedures are thus necessary to minimize contamination of samples at a typical ambient Hg concentration, which commonly is at the nanogram-per-liter level. Because contamination problems during sampling and storage have been reported widely, much attention has been paid to decontamination of the material, cleaning procedures, and appropriate storage of the samples [1,2].

Rigorous cleaning procedures have been established for all laboratory ware including sampling vials and other equipment that comes into contact with samples. There are various different cleaning procedures available, but they all generally include several acid baths and intermediate rinses with Hg-free deionized water or double distilled water, with storage in a designated Hg-free area, preferably sealed in clean plastic bags [1]. The importance of the choice of the material for sampling storage has been previously discussed [2]. For example, water samples can be stored in polytetrafluoroethylene (PTFE, Teflon^®^), glass or polyethylene terephthalate (PET) bottles (see ref [1] and references therein). Quartz or borosilicate glass bottles minimize the loss of Hg(II) [3] and are suitable for MeHg solution storage [4]. For extremely low concentrations, as found in seawater, PTFE presents the lowest risk for contamination [1]. However, PTFE bottles are more expensive than glass bottles and not all laboratories can afford them.

Here, we provide a complete list of materials needed to achieve effective sampling of the typically low ambient Hg levels found in freshwaters. To prevent contamination, we describe a protocol for equipment cleaning and sample processing. As there is no consensus on reporting IHg and MeHg concentration from filtered or unfiltered water fractions, we give details for collecting both water fractions: filtered (dissolved) and unfiltered (total). Although detailed protocols for collection of water with low Hg levels are available (e.g. https://water.usgs.gov/owq/FieldManual/chapter5/pdf/5.6.4.B_v1.0.pdf), here we describe an updated, simple and didactic protocol for collecting samples from freshwater ecosystems for Hg analyses. Furthermore, as Hg binds strongly to organic matter, we include a procedure to collect samples for determining dissolved and total organic carbon concentration (DOC and TOC) as well as DOM composition. Consequently, this protocol can be extremely helpful for non-experts in Hg sampling and analysis, as well as for all Hg research focusing on the role of organic matter composition related to Hg concentrations in the water [[5], [6], [7], [8], [9]]. It is noteworthy to mention that this protocol does not provide guidelines on the sampling design. The users of this protocol are encouraged to plan their sampling considering the optimal site location (e.g. deepest point of lake, distance from shoreline, etc.), depth (e.g. hypolimnion, epilimnion, etc), number of replicates, number of times the sampling will be performed in a year, etc. It is also important to highlight that because the choice of material used to sample with is glass (instead of PTFE), this protocol is not suitable for seawaters and water with ultra trace Hg levels (i.e. below 0.01 ng L^−1^ for IHg and 0.002 ng L^−1^ for MeHg).

Four different water samples will be collected following the protocol described here:

1
  1 Total-Hg, IHg and MeHg (unfiltered): 250 mL amber borosilicate glass bottle.
  2 Total-Hg, IHg and MeHg (filtered): 250 mL amber borosilicate glass bottle.
  3 TOC (unfiltered): 60 mL glass vial.
  4 DOC, fluorescence spectroscopy, anions and cations (filtered): 60 mL glass vial.

## List of materials (Fig. 1)

• 60 mL glass vials (EPA, VWR, ref: 548-0156) and screw white caps in polypropylene and polytetrafluoroethylene (PTFE, VWR, ref: 548-0871). Half of the bottles should be labeled as filtered, the other half as unfiltered.
• 250 mL amber borosilicate bottles (Boston Round Glass Bottles, Unlined, Standard, Thomas Scientific, ref: D0154-8). (Note that Teflon^®^ bottles may be also used, although they are more expensive). Half of the bottles should be labeled as filtered, the other half as unfiltered.
• Filters: Sterivex-HV; 0.45 μm (PVDF, ref: SVHV01015, Merck).
• Syringes: Single-use syringes, 2-piece, NORM-JECT^®^ (50 mL VWR).
• Hydrochloric acid (HCl, 33–36%, Ultrex II ultrapure reagent, J.T. Baker, Phillipsburg, NJ, USA). Prepare also a 2 M solution with this acid.
• Tips: 50 −1000 μL (VWR, ref: 612–5756).
• Nitrile gloves: (VWR, for reference number check your size).
• Cooling box/Styrofoam box with cooling elements.
• Plastic bags (zip sealed) for sample storage (Minigrip^®^ bags, PE, VWR, ref: MINIITM024243).
• Pipette (50−1000 μL)

The above list of materials accounts for materials needed for one sample replicate. Multiply accordingly to sample size, based on the number of study sites and an additional minimum of 3 blanks.

## Cleaning

Ultra clean procedures are required to prevent contamination of samples. Wear clean gloves and avoid breathing directly over the samples.

The cleaning procedure for the amber borosilicate bottles used to store water and analyse mercury species should be carried out in a series of three baths:

• A soap Extran^®^ bath for 1 h under sonification* and then rinsed with MilliQ water.
• 2-h sonification* in a 10% (v:v) HNO_3_ (Emsure^®^, Merck, ref: 1004562500) bath, conducted a second time after changing the acid bath and rinsing with MilliQ water.
• 2-h sonification* in a 10% (v:v) HCl (Emsure^®^, Merck, ref: 1003172500) bath followed by a MilliQ water rinse.

*If an ultrasonic bath sonicator is not available, leave the laboratory ware in each of the baths for 48 h.

60 mL glass vials used to store water and analyse DOC and TOC should be muffled at 550 °C for 1 h.

The cleaning procedure for the screw caps used to store water and analyse mercury species and DOC/TOC concentration should follow the same procedure as for the bottles, but the soap and the acid baths should be more diluted in order to avoid any damage:

• A soap Extran^®^ bath diluted 10 times (100 mL of soap and 900 mL of MilliQ) for 1 h under sonification and then rinsed with MilliQ water.
• 2-h sonification* in a 3% (v:v) HNO_3_ (Emsure^®^, Merck, ref: 1004562500) bath, conducted a second time after changing the acid and rinsing with MilliQ water.
• 2-h sonification* in a 3% HCl (Emsure^®^, Merck, ref: 1003172500) bath followed by a MilliQ water rinse.

*If an ultrasonic bath sonicator is not available, leave the screw caps in each of the baths for 48 h.

## Sampling procedure

This protocol was provided to 30 early career researchers, who were non-experts in Hg sampling and analysis, to collect surface river waters across a wide geographical scale in Europe [9].

1 Put on nitrile gloves.
2 Label all bottles.
3 Condition bottles to collect UN-filtered water samples for TOC and total-Hg, IHg and MeHg:
  a Take the 250 mL brown glass bottle labeled “*Your sample name* Mercury unfiltered” and the 60 mL glass vial “*Your sample name* TOC unfiltered”. These are your UN-filtered water samples.
  b Bottle Conditioning: fill a bit more than half of the brown glass bottle labeled “*Your sample name* Mercury unfiltered” with site water sample. The collection of the surface microlayer should be avoided by immersing the bottle closed. Shake vigorously and discard the water downstream or away from the sampling site. Repeat three times. Do the same procedure with the 60 mL glass vial labeled “Your *sample name* TOC unfiltered”.
4 Collect unfiltered total-Hg and MeHg sample: Fill the pre-conditioned brown glass bottle “*sample name* Mercury unfiltered” (not completely, leave about 0.5 cm without water). Add 250 μL of HCl (concentrated) acid (Ultrex II ultrapure reagent, J.T. Baker). Close the bottle with the screw cap lid and shake gently. You have now collected UN-filtered water for total-Hg, IHg and MeHg measurements. Keep it cold and dark in the cooling box in the field, and store it in the fridge until the samples get measured or send it immediately for delivery if no more samples need to be collected (Fig. 2).

**Fig. 1:**
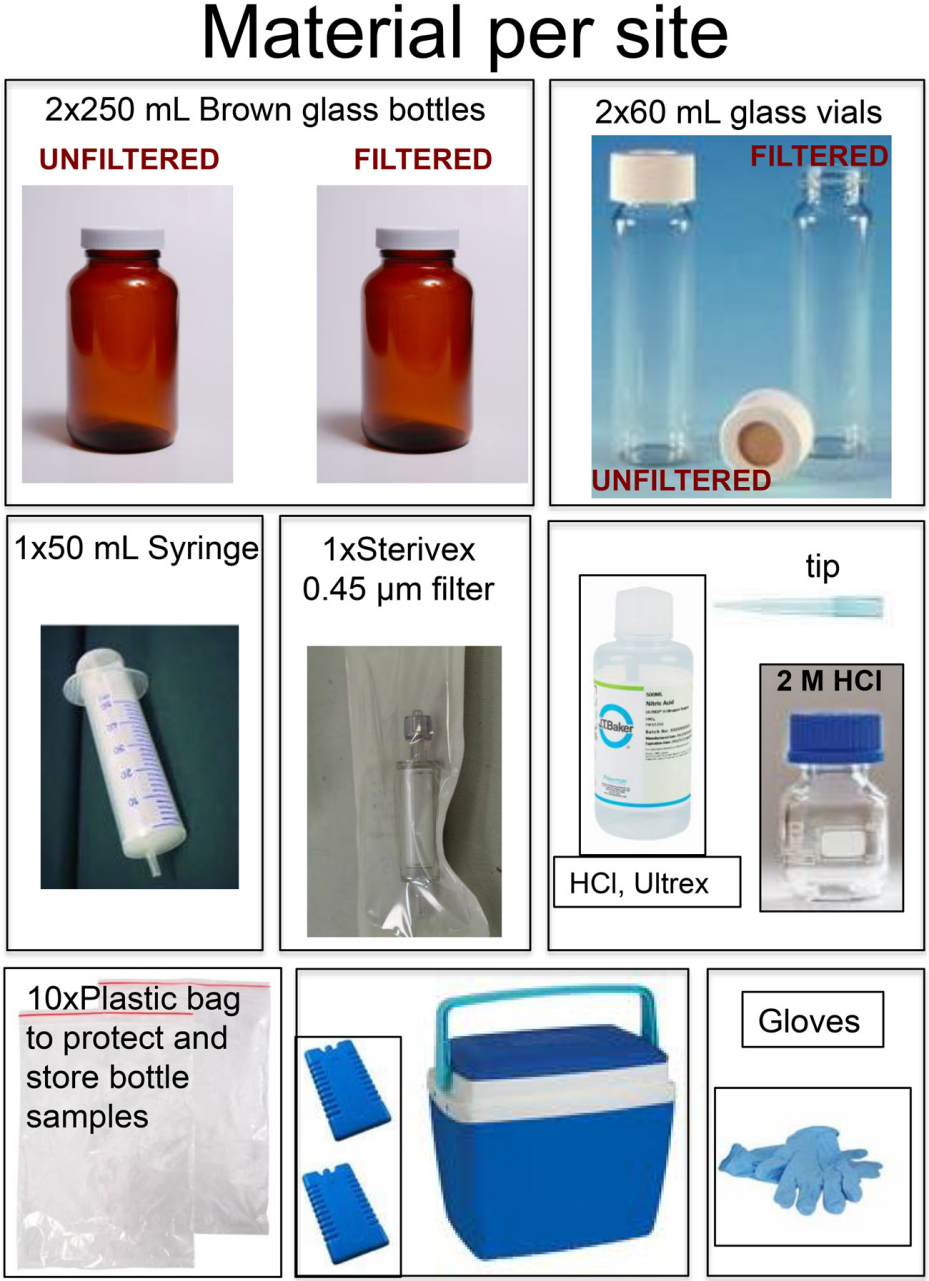
Summary of the material needed for one sample replicate.

**Fig. 2:**
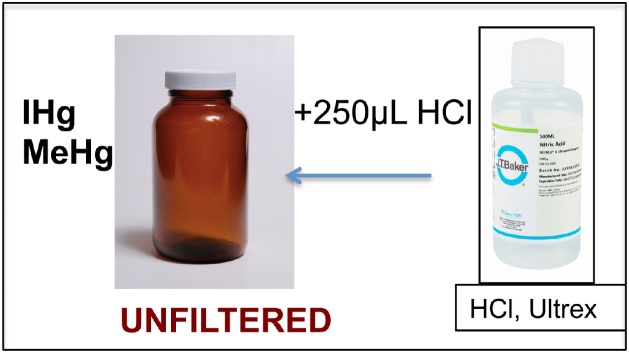
Preserving unfiltered water sample for mercury species (total-Hg, IHg and MeHg) analyses.
5 Collect TOC sample: Fill completely (without bubbles, headspace free) the 60 mL glass vial “*Your sample name* TOC unfiltered” once more (after bottle conditioning). This is now the sample for TOC (unfiltered). Keep it cold and in dark in the field and store it in the fridge in the lab or send it immediately by mail currier if no more samples need to be collected. TOC samples should be acidified with HCl 2 M (100 μL) and preserved at 4 °C until analysed (Fig. 3).

**Fig. 3:**
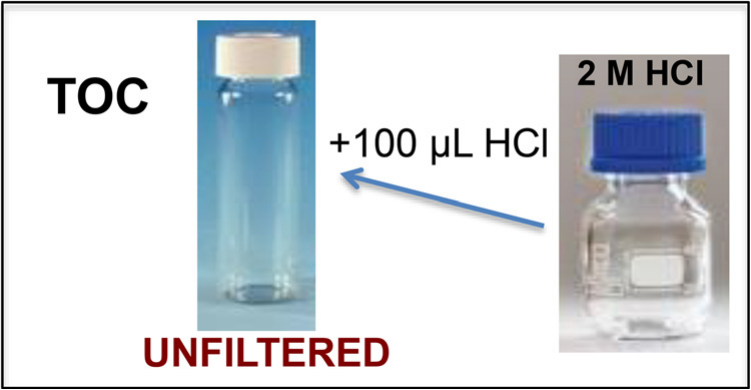
Preserving unfiltered water sample for TOC analyses.
6 Collect DOC sample:
  a. Rinse the syringe: Take the syringe. Rinse it three times with site water.
  b. Rinse the filter: Fill your syringe. Connect the Sterivex filter to the syringe. Start filtering and waste the first 20 mL of sampling water.
  c. Rinse the 60 mL glass vial labeled “*Your sample name* DOC filtered”: Put the remaining 30 mL in the 60 mL glass vial labeled “*Your sample name* DOC filtered”. Shake the vial vigorously and waste the filtered water. Repeat this two more times (for a total of three).
  d. Collect the sample: Filter site water and fill (without bubbles, headspace free) the 60 mL glass vial “*Your sample name* DOC filtered” that you have previously rinsed. You have now collected filtered water sample for DOC, fluorescence, anions, cations and any ancillary parameter of the dissolved fraction. Keep it cold and dark in the field and store it in the fridge in the lab or send it immediately by mail if no more samples need to be collected. DOC samples should be acidified with HCl 2 M (100 μL) prior to analysis. In the laboratory acidify to a pH of 3.5 and preserve at 4 °C until analysed. Before acidifying, safe a subsample for fluorescence, anions and cations measurements.
7 Collect filtered water for total-Hg, IHg and MeHg measurements:
  a. Take your syringe and Sterivex filter, filter 60 mL of sample and put it in the brown glass bottle “*Your sample name* Mercury filtered”. Shake the brown bottle vigorously and waste the water. Repeat two more times (for a total of three).
  b. Collect sample water and filter 250 mL into the brown glass bottle labeled “*Your sample name* Mercury filtered” (avoid the surface microlayer by immersing the syringe at least 10 cm below the surface). Add 250 μL of HCl concentrated acid (33–36%, Ultrex II ultrapure reagent, J.T. Baker, Phillipsburg, NJ, USA). (In Bravo et al., [9]) this acid was provided in cleaned gas chromatography glass vial). You have collected the water sample for measurements of filtered total-Hg, IHg and MeHg.
8 Double-bag all sample containers individually in zip-sealed (Minigrip^®^) plastic bags. Keep it cold and dark in the field and store it in the fridge when back to the laboratory. Rebag all samples until processing and preservation. Store and transport all sampling and processing equipment and supplies in a clean plastic container. Use packing foam if necessary to ensure that bottles are securely protected during transport.

## Summary of the samples collected (Fig. 4)

**Fig. 4:**
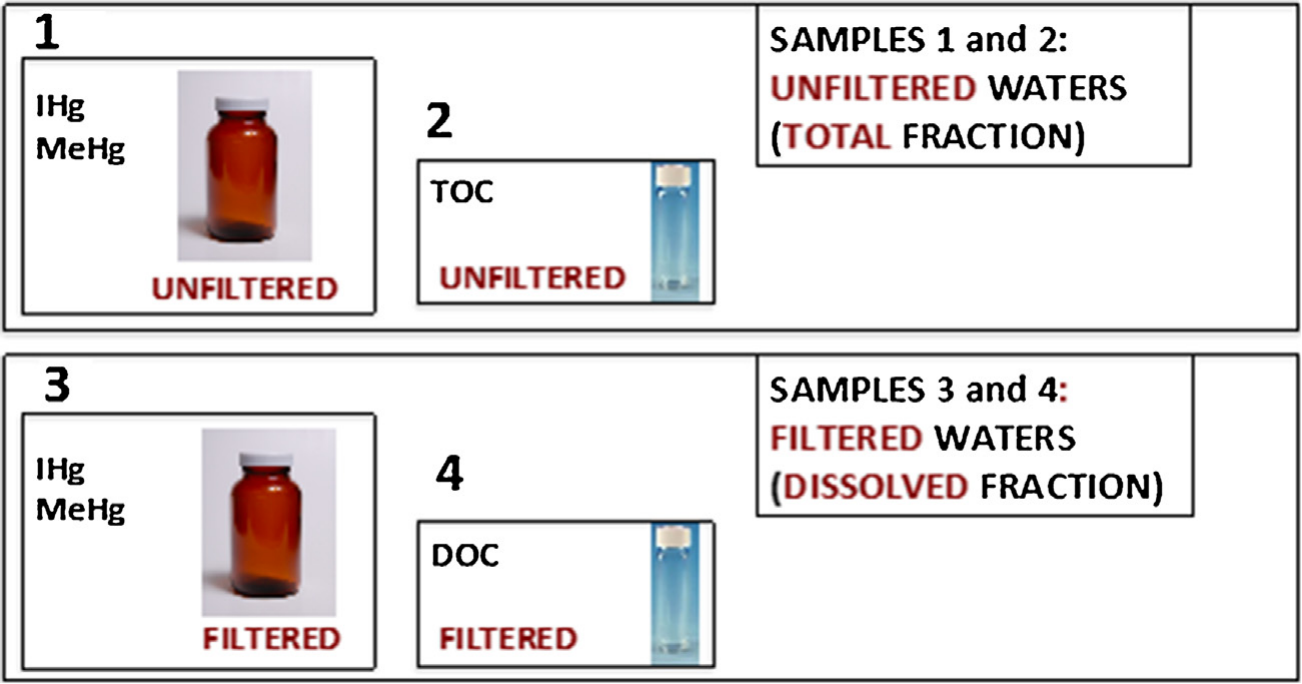
Summary of the samples collected. Besides these, you need to collect your blanks.

## Preparing the blanks

**Fig. 5:**
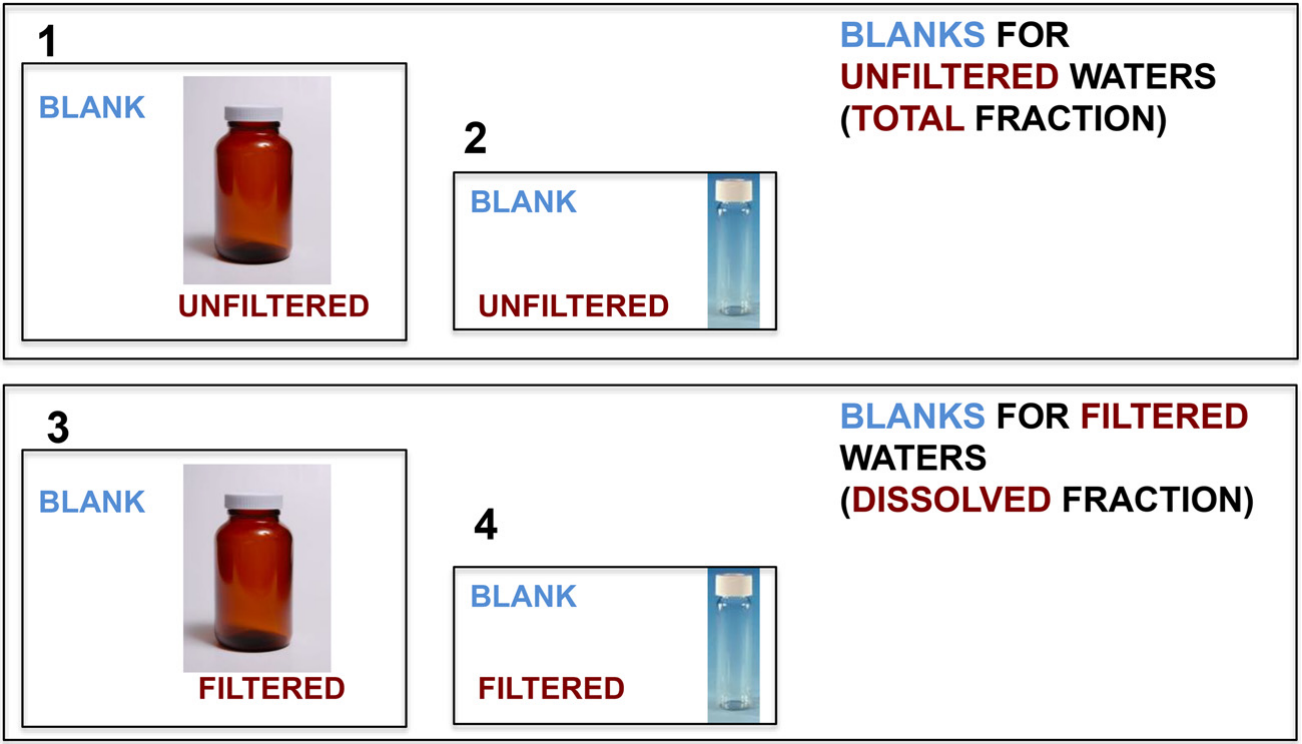
Summary of the blanks needed to ensure the quality of the sampling.

Collect an initial field blank to evaluate the potential for contamination associated with the field methods, materials used, and sampling environment. Subsequent field blanks should be collected to address field-site concerns, the sampling time frame, and data-quality requirements. Field blanks are processed in the same manner and under the same environmental conditions as environmental samples. Follow steps 1–7 but use MilliQ water instead. Repeat this five times per sampling campaign in order to have several sample blank-replicates (Fig. 5).

## Analytical methods

**Fig. 6:**
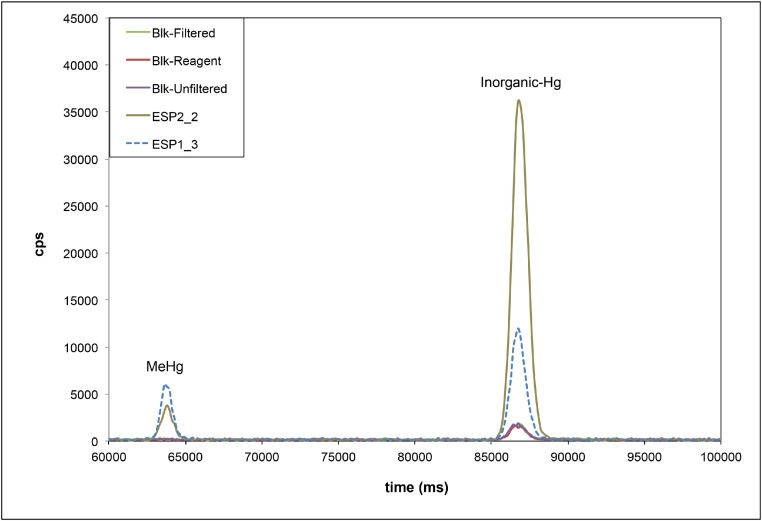
Mass chromatograms of two samples with contrasting methylmercury and inorganic mercury concentrations and different blank (Blk) samples that tested: i) the reagents (Blk-Reagent); ii) the sampling, handling, storage and reagents (Blk-Unfiltered); and iii) the sampling, handling, filtering, storage and reagents (Blk-Filtered). The lines represent the average of 3 replicate samples (each of them calculated by three analytical replicates).

Different methods can be used to measure IHg and MeHg [10]. IHg and MeHg concentrations were measured using species-specific isotope dilution and capillary gas chromatography (Trace GC Ultra, Thermo Fisher, Waltham, MA, USA equipped with a TriPlus RSH auto-sampler) hyphenated to a inductively coupled plasma mass spectrometer (Thermo X Series 2) [11,12]. The detection limits of this method are 0.02 ng L^−1^ for IHg and 0.005 ng L^−1^ for MeHg. Total-Hg concentration was calculated by adding IHg and MeHg concentrations. The measurement error (calculated by analyzing each sample three times) was less than 2.6% and 3.8% for IHg and MeHg concentrations, respectively [9] (Fig. 6).

## Quality assurance and quality control (QA/QC)

Besides the strict control of the blanks, the protocol and analytical procedure has to be coupled to an extensive Quality Assurance and Quality Control (QA/QC) procedure, involving replicate tests, certified reference materials and, if possible, inter-laboratory comparisons.

a) To calculate the measurement error (precision), analyse each sample three times. Be sure you have less than 15% for IHg and MeHg concentrations, respectively.
b) To estimate the accuracy, perform repeated analyses of certified reference materials (CRM, e.g. ORMS-5, NRNCC https://www.nrc-cnrc.gc.ca/eng/solutions/advisory/crm/certificates/orms_5.html) and verify that your values are always within the range of concentration given for CRM (e.g. ORMS-5, THg = 26.2 ± 1.3 pg g^−1^).
c) By measuring the same sample at two different laboratories, you can perform an inter-laboratory comparison and identify any pitfall associated with a particular laboratory or operator. It is thus recommended to measure at least three samples in two different laboratories (analytical platforms).

## Green analytical chemistry

Throughout this protocol we encourage the use of green analytical chemistry. The main goal of green analytical chemistry is to achieve a more eco-friendly analysis in laboratories. This can be done through different strategies and techniques, replace toxic reagents, and modify or replace analytical methods and techniques with safer ones, making it possible to reduce the amounts of reagents consumed and waste generated [13]. In the cleaning protocol here described, we used three reagents: soap Extran^®^, HNO_3_ and HCl, which are unavoidable to ensure that all laboratory ware is free from contamination. These cleaning reagents need to be handled with care because of their toxicity (oral, dermal, inhalation), skin irritation, eye irritation and the risk of corrosion (read carefully the safety documentation of the product). However, treated with care, the waste is relatively easy to treat and can be simply neutralized [14]. Handling acid baths with care and precautions extends their life-time and thus reduces the amount of waste. Therefore, make sure that all the tubes put in the baths have been previously washed with the soap Extran^®^ and rinsed with MilliQ. Keep these baths designated for low level Hg analysis. This reduces the costs and the environmental impact of the study. Glass bottles and plastic syringes used for sampling can be re-used several times until they break. The unavoidable use of plastic pipette tips and Sterivex-HV filters are the main waste products.

The green analytical chemistry of total-Hg, IHg and MeHg measurements can vary depending on the analytical method used. In particular, in Bravo et al. [9], and according to Rodriguez Martín-Doimeadios et al., [12], we used low amounts of isooctane (< 700 μL per sample), acetic acid-sodium acetate buffer (5 mL per sample), tetrapropylborate (50 μL per sample) and enriched Hg stable isotopes (at environmental relevant concentrations). Solvents are one of the most critical reagents for green analytical chemistry [15]. Isooctane has been listed as a usable solvent in the Pfizer solvent selection guide [15].

## Concluding remarks

This protocol is designed for non-Hg experts, as well as for those with experience with Hg, and should help national monitoring agencies developing routine Hg monitoring programs to report the effectiveness of the Minamata Convention. This protocol is a novel contribution since it combines the concepts required for sample collection and measurement of filtered and unfiltered Hg species, DOC and DOM in a single easy protocol, which summarizes and unifies existing protocols in the bibliography. This method provides non-expert users with a basic platform, upon which users can improve and optimize. The detailed protocol described above has been proved to be successful when used by a group of 30 researchers with no prior experience with the sampling and analysis of IHg or MeHg in freshwater systems [9].
